# Case Report: Rhabdomyolysis in a Patient with COVID-19: A Proposed Diagnostic-Therapeutic Algorithm

**DOI:** 10.4269/ajtmh.20-0692

**Published:** 2020-07-29

**Authors:** José Gabriel Solís, Alejandra Esquivel Pineda, Paolo Alberti Minutti, Alejandra Albarrán Sánchez

**Affiliations:** Internal Medicine Department, Centro Médico Nacional Siglo XXI, Instituto Mexicano del Seguro Social (IMSS), Mexico City, Mexico

## Abstract

COVID-19 represents the greatest health challenge of modern years. The spectrum of illness comprises respiratory and non-respiratory manifestations. We report the case of an adult man with COVID-19 who presented with rhabdomyolysis as a principal extrapulmonary manifestation. Our patient presented with dyspnea, fever, and muscle pain. After a comprehensive approach, the diagnosis of COVID-19 and rhabdomyolysis was made. He developed acute kidney injury requiring renal replacement therapy without reversibility, despite optimal treatment. We performed a literature search for similar cases, discuss the potential mechanisms implied, and propose a diagnostic-therapeutic algorithm.

## INTRODUCTION

COVID-19 represents the greatest healthcare challenge of modern years. SARS-CoV-2 has infected more than 13 million people and caused more than 500,000 deaths worldwide.^1^ The spectrum of illness ranges from a mild respiratory infection to severe pneumonia and acute respiratory distress syndrome. There is a wide range of extrapulmonary manifestations such as renal, cardiac, and neurological.^2^

We report the case of a patient with confirmed SARS-CoV-2 infection who presented with rhabdomyolysis as a cardinal manifestation, discuss the possible mechanisms, and propose a diagnostic-therapeutic algorithm.

## CASE PRESENTATION

A 46-year-old man presented to the emergency department with a respiratory illness of 5-day evolution characterized by cough, fever, dyspnea, and generalized muscle pain. His medical history was remarkable for chronic myeloid leukemia treated with imatinib, being its last dose 3 months before hospitalization, with an optimal response. He denied recent trauma, use of drugs, or exposure to toxins. On admission, the patient was tachycardiac, tachypneic, and hypoxemic. Blood pressure and temperature were normal. Physical examination revealed bilateral pulmonary rales and generalized muscle pain.

**Figure 1.**
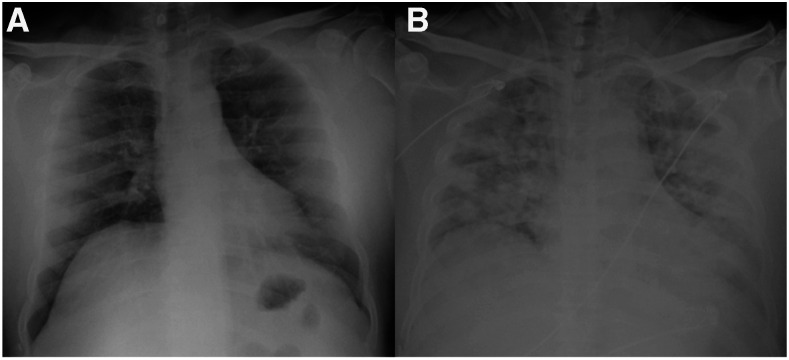
Radiographic evolution. (**A**) Chest X-ray at hospitalization showing bilateral ground-glass opacities with peripheral predominance. (**B**) Chest X-ray on day 7 showing radiographic progression, with diffuse bilateral alveolar occupation and consolidations.

His chest X-ray showed bilateral and diffuse ground-glass opacities with a predominantly peripheral distribution (Figure 1A). Laboratory tests revealed grade 3 acute kidney injury (AKI) with a creatinine level of 11 mg/dL (basal value 0.7 mg/dL); increased blood levels of creatine kinase (CK) (> 400,000 U/L), lactate dehydrogenase (LDH), aspartate aminotransferase, alanine aminotransferase; and electrolyte disturbances with hyperkalemia, hyperphosphatemia, hypocalcemia, and severe metabolic acidosis. Also, he had lymphopenia, moderate thrombocytopenia, and elevated C-reactive protein and ferritin. His coagulation panel showed elevated fibrinogen levels and D-dimer. His urinary volume in the first 12 hours of hospitalization was 20 mL. Urinalysis revealed dark urine, urine dipstick positive for hemoglobin, and a normal sediment, compatible with myoglobinuria.

Nasopharyngeal swab with real-time reverse-transcriptase polymerase chain reaction (RT-PCR) for SARS-CoV-2 was positive. Real-time reverse-transcriptase PCR for influenza virus was negative. Additional investigations included fourth-generation ELISA for HIV, hepatitis B surface antigen, and serologic tests for hepatitis C virus, cytomegalovirus, herpes simplex, rubeola, toxoplasma, and Epstein–Barr virus, all of which were negative.

The diagnosis of COVID-19 and severe rhabdomyolysis complicated with AKI was made. The patient was treated with supplemental oxygen therapy and azithromycin. Treatment with intravenous solutions and sodium bicarbonate was administrated without response, persisting with anuria, and developing uremic encephalopathy. Continuous renal replacement therapy was instituted with a continuous veno-venous hemodiafiltration modality.

**Figure 2.**
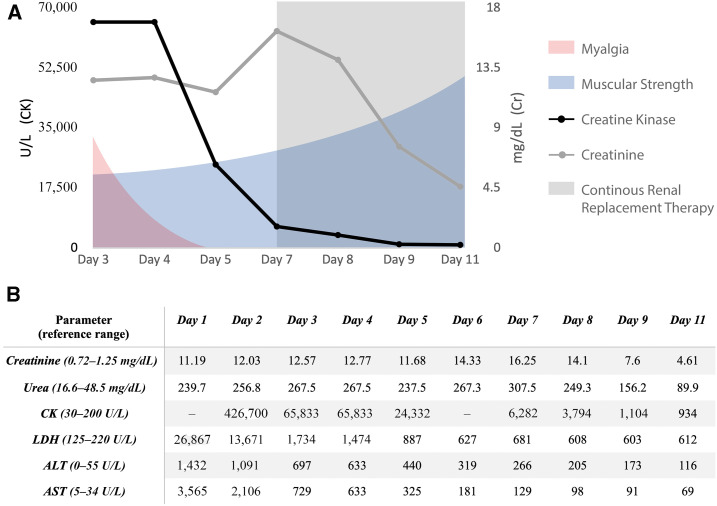
Clinical and biochemical evolution. (**A**) Clinical evolution according to myalgias and muscular strength, and their correlation with muscle enzymes and kidney function during hospitalization. (**B**) Progressive reduction of CK and creatinine levels during hospitalization. Kidney function did not return to baseline. ALT = alanine aminotransferase; AST = aspartate aminotransferase; CK = creatine kinase; LDH = lactate dehydrogenase.

On day 5 of hospitalization, the patient developed fever and elevated procalcitonin levels. A new chest X-ray showed new infiltrates and bilateral consolidations, compatible with disease progression or bacterial superinfection (Figure 1B). Broad-spectrum antibiotic therapy was initially administered and suspended after sputum and blood cultures were negative. The patient showed progressive reduction in muscle pain, improvement of strength, decrease in muscle enzyme levels (Figure 2), resolution of electrolyte disorders, and stabilization of kidney function. Unfortunately, he developed further respiratory impairment and died during his hospitalization.

## DISCUSSION

Rhabdomyolysis is a life-threatening entity characterized by rapid destruction of skeletal muscle fibers, causing the release of toxic intracellular components into the bloodstream. The diagnosis of this condition requires a high index of suspicion. The most accepted diagnostic criterion is an elevation of CK greater than 1,000 U/L, considering severe rhabdomyolysis with a cutoff value of 5,000–15,000 U/L.^3^

The classic clinical triad myalgia, muscle weakness and, pigmenturia is present in less than 10% of cases. In addition to increased amounts of serum CK, other biochemical abnormalities include elevated transaminases, LDH and myoglobin, myoglobinuria, and electrolyte or acid–base disturbances, such as hyperkalemia, hyperphosphatemia, hypocalcemia, hyperuricemia, and metabolic acidosis.^4^

The development of rhabdomyolysis is associated with a wide variety of conditions such as trauma, drugs, toxins, autoimmune myopathies and viral infections. Although viral infection is the most frequent cause in children, it is seldom reported in adults. Influenza virus is the most common viral etiology of rhabdomyolysis; however, many others have been described, including enterovirus, HIV, parainfluenza, adenovirus, cytomegalovirus, coxsackievirus, Epstein-Barr, herpes simplex, echovirus, varicella-zoster, and dengue virus.^5^

SARS-CoV-2 has shown a wide variability of systemic manifestations. Rhabdomyolysis has been described anecdotally with just a few cases reported so far.^6–9^ In addition, there were two patients with rhabdomyolysis in China’s first multicenter cohort that included a total of 1,099 patients.^10^ Rhabdomyolysis can develop either as a first manifestation or as a complication of the disease, independently of the presence of respiratory symptoms. This highlights the need of a high index of suspicion, especially during the actual pandemic.

Rhabdomyolysis has been previously described in infections caused by other beta-coronaviruses. It developed in up to 10% of patients with severe acute respiratory syndrome (SARS)^11^ and in 14% of Middle East respiratory syndrome (MERS).^12^ An important issue is that they all received high doses of intravenous steroid and some required neuromuscular blocking agents, contributing factors that were absent in our patient.

Physiopathology in viral myositis is not entirely known, and the mechanism by which SARS-CoV-2 can cause rhabdomyolysis has not been studied. Direct viral invasion of muscle tissue and toxicity mediated by cytokines or immunological cross-reactivity have been proposed.^13^ In a recent study from Brazil in which 10 autopsies from patients with COVID-19 were performed, histological analysis of skeletal muscle showed myositis in 60% and necrotic fibers in 80% of the patients.^14^ However, in patients with SARS and rhabdomyolysis, muscle biopsies did not show inflammatory cell infiltration.^15^

AKI is the most important complication of rhabdomyolysis and occurs in 13–50% of the cases. AKI can be secondary to direct tubular injury, tubular obstruction, and intrarenal vasoconstriction.^16^ The frequency of AKI in COVID-19 is variable, ranging from 5% to 60%; however, it has a clear association with mortality.^17^ SARS-CoV-2 can cause AKI through diverse mechanisms including virus-mediated injury, cytokine storm, angiotensin II pathway activation, dysregulation of the complement pathway, hypercoagulation, and microangiopathy, all of which could have contributed in our patient.^18^ Also, tubular toxicity from rhabdomyolysis has been considered before.^19^ In a study of 26 autopsies, the histopathological analysis of 3 patients revealed pigment casts in the renal interstitium, which correlated with elevated levels of CK, probably related to rhabdomyolysis.^20^

The management of patients with rhabdomyolysis in COVID-19 is challenging. The underlying cause of muscle injury must be identified and treated, which is difficult in patients with COVID-19 because there is no specific therapy. Other factors that can contribute to muscle injury should be investigated, such as coinfections or drugs. In this case, although our patient was previously treated with imatinib, there was no time relationship with the development of rhabdomyolysis.

**Figure 3.**
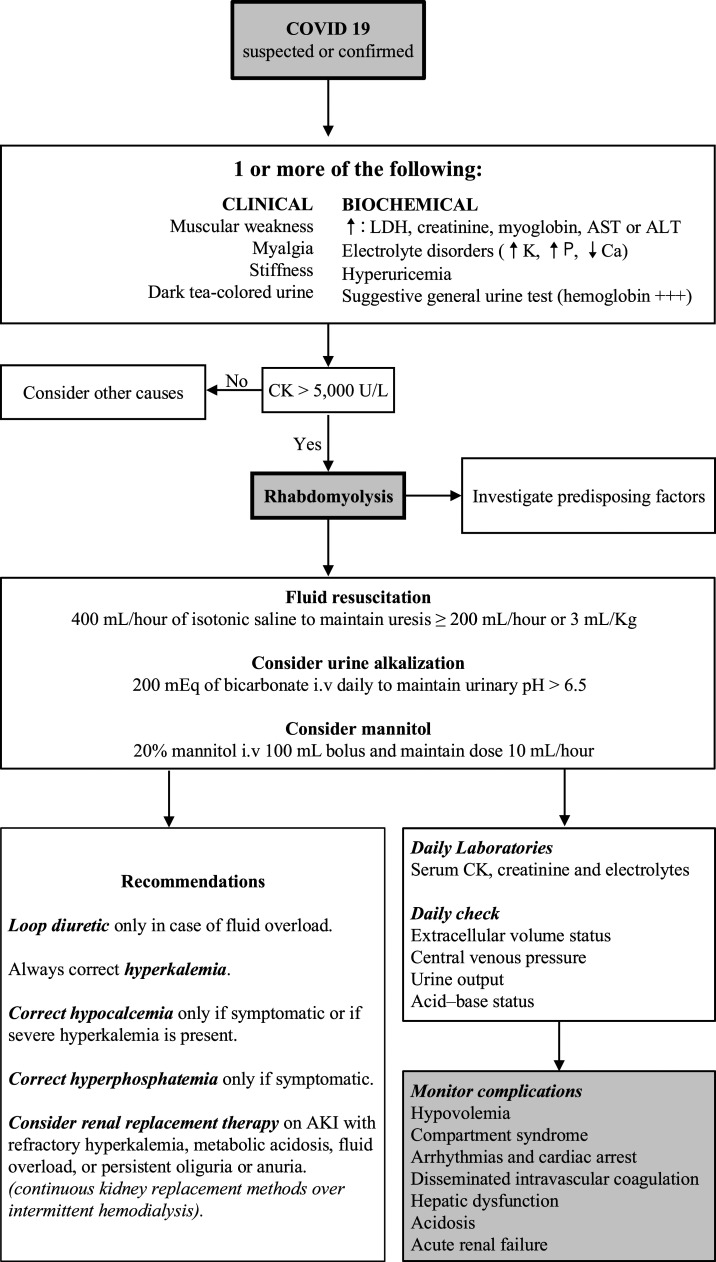
Algorithm for the diagnosis and management of rhabdomyolysis in COVID-19. ALT = alanine aminotransferase; AST = aspartate aminotransferase; CK = creatine kinase; LDH = lactate dehydrogenase.

Fluid replacement is the keystone of rhabdomyolysis treatment. Other therapies include the use of bicarbonate or mannitol.^4^ Electrolyte disorders and AKI must be detected and treated if required. The indication of renal replacement therapy is based on patients AKI and not based on the levels of CK. Although there is no specific modality recommended in rhabdomyolysis, continuous veno-venous hemodiafiltration with high permeability membranes seems to be more effective.^21^ An algorithm for the detection and management of rhabdomyolysis in COVID-19 is proposed (Figure 3).

## CONCLUSION

Rhabdomyolysis is a rare and probably underdiagnosed complication of SARS-CoV-2 infection. It should be suspected in patients with disproportionate myalgia, muscle weakness, dark urine, unexplained hyperkalemia, or metabolic acidosis. Acute kidney injury can develop as a complication that has implications in treatment and mortality. We recommend measuring CK levels in COVID-19 patients with suggestive clinical or analytical alterations, especially in those with AKI, in which it can be a pathophysiological mechanism that requires early and aggressive treatment to prevent chronic kidney damage or death.
